# Sanitary Condition of Philadelphia

**Published:** 1876-05

**Authors:** William Pepper

**Affiliations:** Medical Director


### Sanitary Condition of Philadelphia.

We have received the following circular to which we direct the attention of our readers:

UNITED STATES CENTENNIAL COMMISSION. BUREAU OF MEDICAL SERVICE.

Owing to the very large number of persons who contemplate a visit to Philadelphia during the coming summer, it seems important that the utmost publicity should be given to all facts bearing on the sanitary condition of the city.

The following statistics, which have been obtained from the most authentic sources accessible, represent the mortality in some of the chief cities of the world during the past four or five years :—

| | NUMBER OF YEARS. | AVERAGE POPULATION. | AVERAGE TOTAL MORTALITY. | AVERAGE DEATH RATE PER THOUSAND. |
|---|---|---|---|---|
| Vienna.... ............... | 5 | 648,560 | 20,424 | 31.42 |
| New York................. | 5 | 994,458 | 29,601 | 29.93 |
| Berlin.................... | 4 | 950,000 | 28,420 | 29.91 |
| London................... | 5 | 3,284,488 | 76,741 | 23.33 |
| Paris..................... | 4 | 1,851,792 | 42,724 | 23.06 |
| Philadelphia............... | 5 | 744,831 | 16,573 | 22.27 |

While thus showing an average rate of mortality more favorable than that found in any other city containing over 500,000 inhabitants, Philadelphia has recently (1874)attained a degree of healthfulness almost unparalleled, viz.: with a population at that time of 775,000, the number of deaths was but 14,966, giving a death rate of only 19.3 per thousand. These very fávorable results are largely due to the abundant and cheap water-supply, and to the opportunities given, even to the poorest citizens, for the enjoyment of pure country air in the great Fairmount Park, which contains 2,991 acres. The extent to which this is valued by the citizens may be inferred from the fact that during the year 1875, the Park was visited by over eleven million persons.

The most powerful influence of all, however, is the absence of that overcrowding of the population, which is the most fruitful source of sickness and death in many quarters of nearly all other large cities. This will be more clearly comprehended when it is remembered that the 817,488 inhabitants of Philadelphia are spread over an area of 129⅛ square miles, which are traversed by more than one thousand miles of streets and roads ; and that the city eontains, in addition to other kinds of buildings, 143,000 dwelling-houses occupied by families,—a number exceeding by over 40,000 that of any other city in America.

The climate of Philadelphia is also, on the whole, a favorable one, although presenting many of the pecnliarities common to inland localities. The mean annual temperature of the last ten years is 53.73° Fahrenheit ; the average annual rain-fall is about forty-five inches.

The following table exhibits the mean temperature of each month for the past ten years, showing that the range is far less extreme than is found in many other less favorably situated tocalities :—

Mean Temperature (Fahrenheit) of each Month durin the past Ten Years.

| | | | | | |
|---|---|---|---|---|---|
| January ................. | 32.72° | F. | July.................... | 78.74° | F. |
| February............. .. | 33.12 | " | August........... ...... | 75.92 | " |
| March................... | 39.16 | " | September............... | 67.72 | " |
| April.................... | 53.36 | " | October ................ | 56.03 | " |
| May........ ............ | 63.24 | " | November............... | 43.34 | " |
| June.................... | 73.54 | " | December............... | 33.92 | " |

It is thus seen that only during the months of June, July, and August does the mean temperature rise to a high point. During this period there are very rarely any prevailing epidemic diseases ; and the chief mortality occurs among children, especially among the poorer classes.

The health of Philadelphia at present is unusually good. Timely efforts have been made to secure an abundant water-supply to meet the great increase in the demand which must be expected this summer as compared with previous years. Constant watchfulness will be exercised by the authorities to maintain cleanliness, and to avoid or remove every possible cause of disease.

Within the Exhibition grounds a rigid sanitary inspection will be maintained, under the control of the Bureau of Medical Service ; and thus a guarantee will be afforded that no cause of infection or disease will be allowed to occur through neglect of this important duty.

The object of this circular has been to call attention to the unusual sanitary advantages of Philadelphia, and to the preparations which have been made to ensure the highest possible degree of healthfulness during the approaching Exhibition season. It is proposed to issue at certain intervals other circulars, announcing in an official and accurate manner the sanitary condition of the city, so that entire security may be felt by all who desire to visit the Centennial International Exhibition.

WILLIAM PEPPER, M. D.,

*Medical Director.*

15th April, 1876.

